# Chronic Inflammation as a Link between Periodontitis and Carcinogenesis

**DOI:** 10.1155/2019/1029857

**Published:** 2019-03-27

**Authors:** Anilei Hoare, Cristopher Soto, Victoria Rojas-Celis, Denisse Bravo

**Affiliations:** Oral Microbiology Laboratory, Department of Pathology and Oral Medicine, Faculty of Dentistry, Universidad de Chile, Santiago, Chile

## Abstract

Periodontitis is characterized by a chronic inflammation produced in response to a disease-associated multispecies bacterial community in the subgingival region. Although the inflammatory processes occur locally in the oral cavity, several studies have determined that inflammatory mediators produced during periodontitis, as well as subgingival species and bacterial components, can disseminate from the oral cavity, contributing therefore, to various extraoral diseases like cancer. Interestingly, carcinogenesis associated with periodontal species has been observed in both the oral cavity and in extra oral sites. In this review, several studies were summarized showing a strong association between orodigestive cancers and poor oral health, presence of periodontitis-associated bacteria, tooth loss, and clinical signs of periodontitis. Proinflammatory pathways were also summarized. Such pathways are activated either by mono- or polymicrobial infections, resulting in an increase in the expression of proinflammatory molecules such as IL-6, IL-8, IL-1*β*, and TNF-*α*. In addition, it has been shown that several periodontitis-associated species induce the expression of genes related to cell proliferation, cell cycle, apoptosis, transport, and immune and inflammatory responses. Intriguingly, many of these pathways are linked to carcinogenesis. Among them, the activation of Toll-like receptors (TLRs) and antiapoptotic pathways (such as the PI3K/Akt, JAK/STAT, and MAPK pathways), the reduction of proapoptotic protein expression, the increase in cell migration and invasion, and the enhancement in metastasis are addressed. Considering that periodontitis is a polymicrobial disease, it is likely that mixed species promote carcinogenesis both in the oral cavity and in extra oral tissues and probably—as observed in periodontitis—synergistic and/or antagonistic interactions occur between microbes in the community. To date, a good amount of studies has allowed us to understand how monospecies infections activate pathways involved in tumorigenesis; however, more studies are needed to determine the combined effect of oral species in carcinogenesis.

## 1. Introduction

Periodontal diseases are dysbiotic conditions in the gingival margin, which are characterized by an imbalance between subgingival communities and the host immune response [1]. Such diseases include gingivitis, which is a reversible condition characterized by the inflammation of the gingiva driven by the combined effect of specific microbial taxa. If not treated, gingivitis could progress to periodontitis, characterized by the destruction of supporting tissues of the teeth. From health to gingivitis, to periodontitis, several ecological successions occur in the subgingival microbiome, leading to both an increased biomass and the establishment of distinct dysbiotic communities. Interestingly, not only local effects in the oral cavity have been associated with such disorders but also periodontitis has been largely considered as a risk factor for a number of both oral and systemic diseases [2–5]. Among these, orodigestive cancers are highly influenced by both a direct carcinogenic effect of periodontitis-associated bacteria in either oral cells or in other body sites and inflammatory mediators migrating from the oral cavity [6, 7]. Either way, there is extensive evidence showing that species such as *Porphyromonas gingivalis* (highly abundant and prevalent in periodontitis) and *Fusobacterium nucleatum* (closely interacting with periodontitis-associated species in the disease) directly activate transduction pathways leading to cell transformation [7–12]. Comparatively, less information exists about other periodontitis-associated bacteria.

However, although increasing evidence links periodontitis and carcinogenesis, the fact that periodontitis is a polymicrobial disease has not been well addressed in the context of cancer. This is especially relevant when evaluating the direct carcinogenic effect exerted by oral bacteria, since combined species act locally in oral cells and also migrate from the oral cavity. Thus, more studies evaluating how interbacterial interactions affect carcinogenesis process are needed.

## 2. Periodontal Diseases

Periodontal diseases are associated with chronic inflammation, which affects the supporting tissues of the teeth including the gums or gingival tissue, as well as the periodontal ligament and the alveolar bone in more severe forms of the diseases [13]. Gingivitis is a periodontal disease characterized by local inflammatory processes driven by subgingival bacteria that in most cases do not promote destruction of the tissues and can be reversible. However, clinically, it is considered as the starting point of other periodontal diseases, such as periodontitis [14]. Periodontitis is triggered by an imbalance between resident subgingival microbiota and the inflammatory response of the host that leads to destruction of the supporting tissues of the teeth, even producing the loss of teeth [13]. According to the World Health Organization, between 35% and 50% of the world population are affected by periodontitis [15]. In the United States, the prevalence of gingivitis in children aged between 3 and 11 years is 9-17%, while at puberty, prevalence rises to 70-90% [16] and corresponds to 47% of adult population [17].

### 2.1. Role of Subgingival Communities in the Etiology of Periodontal Diseases

Both gingivitis and periodontitis are driven by bacterial communities interacting with the host immune system and therefore contributing to the inflammation of tissues. Because of the relevance of the bacterial component, different theories have been proposed in order to establish the importance of these subgingival bacterial communities in the etiology of periodontitis. In 1954, it was proposed that the accumulation of microorganisms promotes the release of compounds that produce inflammation in the gingival tissue [18, 19]. This idea eventually evolved into researchers demonstrating that the colonization of certain anaerobic subgingival bacteria, including *P. gingivalis*, *Treponema denticola*, and *Tannerella forsythia*, promoted both the onset and the development of periodontitis [20]. However, different studies that sought to determine the composition of the bacterial community associated with periodontitis managed to determine that these bacteria were not only present in patients with periodontitis but also in periodontally healthy individuals [1].

This was a key point in supporting current theories establishing that it is not the colonization of specific bacteria what triggers the disease, but rather the changes in the relative abundances of specific taxa in the subgingival communities due to dysbiotic processes occurring in subgingival areas that would determine the development of periodontitis. In this context, Marsh [21] created the concept of “ecological catastrophe,” which establishes that the environmental and host factors, such as poor hygiene, inappropriate diets, and use of tobacco and drugs that produce side effects in the immune defense of the patient, select and enrich pathogenic bacteria, and a disease state. The authors described that an increase in bacterial plaque increases local inflammation, which in turn increases the flow of crevicular gingival fluid (CGF), produces bleeding, and provides proteinaceous nutrients, which increase the proliferation of Gram-negative anaerobes [21, 22].

This theory has been supported by several studies aimed at characterizing the microbiome of periodontally healthy individuals and patients with periodontitis [1, 23–25]. Diaz et al. [26] reviewed these studies concluding that different subgingival microbiomes are characteristic of healthy individuals, as well as patients with gingivitis and periodontitis. While most health-associated bacteria are early colonizers of the subgingival biofilm, periodontitis-associated bacteria are mainly late colonizers. In the periodontitis-associated group of bacteria, species such as *Filifactor alocis*, *P. gingivalis*, *Porphyromonas endodontalis*, *T. forsythia*, and *T. denticola* are found in all the 4 studies reviewed. *P. gingivalis* was proposed as a key player among such species (“keystone pathogen”), since Hajishengallis et al. [27] demonstrated that even when it is found in low abundance in healthy individuals, it can promote changes in homeostasis of the normal microbiota, remodeling it towards a harmful microbiota that promotes destruction of tissues and inflammation in *in vivo* models. This concept was refined by Hajishengallis himself in 2012, proposing the polymicrobial synergy and dysbiosis theory (PSD). This theory adds the fact that every component of a symbiotic and synergistic microbiota is relevant in the onset of the disease and not only the periodontitis-associated bacteria. Thus, the whole dysbiotic community will synergistically initiate processes of tissue inflammation, activate production of cytokines, and initiate the recruitment of immune cells [28].

Interestingly, besides having determined both periodontitis and health-associated bacteria, a third group called “core species,” which are equally prevalent and found in the same proportion both in health and periodontitis individuals, was characterized, being *F. nucleatum* the most abundant in this group [1, 24]. *F. nucleatum* plays a central role in the subgingival biofilm, since it physically interacts with other microorganisms in the subgingiva [29]: *P. gingivalis* [30], *Aggregatibacter actinomycetemcomitans* [31], *Prevotella* spp. [32], *Streptococcus gordonii* [33], *Candida albicans* [34], and others [29, 35]. Such close interactions with several species in the biofilm are reflected in the fact that *F. nucleatum* acts as a bridge attaching early colonizers like *Streptococcus* spp. and other facultative species and late colonizers such as *P. gingivalis* [36, 37]. This process is essential for the ecological successions that establish the subgingival plaque and determine the progression of periodontitis [35], in which thousands of species colonize the subgingival area in an ordered manner.

These successions include specific modification of the local environment in the biofilm [38, 39] which select specific groups of bacteria and eventually induce changes in the subgingival bacterial communities that lead to a dysbiotic community able to induce a deregulation of the host inflammatory response and eventually cause chronic inflammation.

### 2.2. Chronic Inflammation Driven by Periodontitis-Associated Bacteria

In the periodontal pocket, the first host responses to the dysbiotic subgingival community are characterized by the infiltration of natural killer (NK) cells, neutrophils, and granulocytes (polymorphonuclear cells) that promote the initial inflammatory response and the subsequent infiltration of lymphocytes to present antigens to dendritic cells [40]. The neutrophils are overwhelmed with the abundance and persistence of microorganisms, being destroyed or undergoing either apoptosis or necrosis as they interact with bacteria within the gingival crevice.

T cells promote a profile characterized by CD8+ and CD4+ cells that generate a proinflammatory medium rich in cytokines such as tumor necrosis factor alpha (TNF-*α*), interleukin- (IL-) 1, IL-4, IL-10, interferon-*γ* (IFN-*γ*), and transforming growth factor *β* (TGF-*β*) [41]. In addition, T CD4+ lymphocytes produce RANK-L, a cytokine that promotes bone resorption [42]. It was also described that T cells contribute to cell-mediated immune responses by stimulating T helper cells such as Th1, Th2, Th9, Th17, and Th22 and the deregulation of this response could be related to the appearance of the disease and its chronicity [43, 44]. On the other hand, B cells produce antibodies against the microorganisms present in the subgingival pocket in order to eliminate them and decrease the local inflammation [44].

In addition to the inflammatory mediators produced by the immune cells, the gingival epithelium also releases other cytokines such as IL-1, IL-8, and TNF-*α*, which in turn promotes the recruitment of macrophages [45]. Concordantly with these studies performed *in vitro*, in periodontitis tissue samples, an increase in mRNA of IL-1*β*, IL-6, IL8, and TNF-*α*, regulated upon activation normal T cell expressed and secreted (RANTES) and monocyte chemotactic protein-1 (MCP-1), was observed, compared to healthy gingiva [46]. In the same context, a higher expression of IL-1*β* was observed in gingival fluid from deeper sites of periodontitis patients [47].

As a consequence of this inflammatory response, ecological changes in the subgingival region occur, which contribute to the ecological successions in the subgingival area that are associated with periodontitis progression. Interestingly, some periodontitis-associated bacteria have been shown to contribute directly to the chronic inflammation by activating specific intracellular pathways.

Because of the polymicrobial nature of periodontitis and considering that interbacterial interactions occurring in the subgingival biofilm contribute to the disease, current models of periodontitis include the study of the effect of multiple species in the stimulation of immune response. Very recently, Herrero et al. [48] showed that the exposure of epithelial and fibroblast cultures to a dysbiotic biofilm increased the expression of IL-6, IL-8, IL-1*β*, TNF-*α*, and MMP-8. In the same context, other studies showed that epithelial cells produce higher cytokine levels when they are exposed to either monospecies or multispecies biofilms [49]. Interestingly, an increased expression of IL-8, C-X-C motif chemokine ligand 3 (CXCL-3), CXCL-1, IL-1, IL-6, colony-stimulating factor 2 (CSF2), and TNF-*α* was observed in cells stimulated with the multispecies biofilms. Similarly, polymicrobial infection (*P. gingivalis*, *T. denticola*, and *T. forsythia*) using a murine calvarial bone model affected the expression of several genes related to cell proliferation, cell cycle, apoptosis, transport, immune response, and inflammatory response. In the proinflammatory context, the cytokines that increased the most were IL-1, IL-6, and TNF-*α*, which are precisely those related to chronic inflammation and chronic bone damage [50].

Nonetheless, despite the fact that multispecies infection constitutes a more realistic model considering the polymicrobial etiology of the disease, many studies using planktonic monospecies bacteria have permitted to determine the contribution of key species to the inflammatory process. For example, studies using *T. denticola* monoinfections have shown that the bacterium can activate Toll-like receptor 5 (TLR5) through the flagellin, the main component of the bacterial flagellum. This interaction leads to an increase in IL-1*β* and TNF-*α* [51]. *T. denticola* can also suppress the action of antimicrobial peptides such as human *β*-defensin 3, regulating the signaling pathway activated by TLR2 [52]. Additionally, works by Tanabe et al. [53] demonstrated that *T. denticola* peptidoglycan induces the secretion of proinflammatory cytokines such as IL-8, IL-6, and TNF-*α*, in murine macrophages, stimulating the production of PGE2 and decreasing their viability. However, *T. denticola* can also counteract the increase of these cytokines, as it has been shown in a study conducted in peripheral blood mononuclear cells, where it was determined that *T. denticola* hydrolyzes IL-1*β*, IL-6, and TNF-*α* through the PrtP complex (dentilisin or chymotrypsin-like protease (CTLP)) [54].

On the other hand, the infection of mice with *T. forsythia* increased levels of IgG and IgM, both markers of immune response activation. Moreover, an increase in CD4+ T lymphocytes was shown [55]. Intriguingly, this bacterium has a glycosylated S layer [56], which is important for the mechanical stabilization and protection of the bacterium. A study by Settem et al. [57] showed that glycosylation of S layer of *T. denticola* can deregulate the immune response by preventing Th17 production, probably inhibiting the recruitment of neutrophils to the site of infection. This effect produces tissue and bone destruction.

A Gram-positive anaerobic bacterium that has been emerging as a periodontitis-associated species is *F. alocis.* Infection of gingival epithelial cells (GECs) by *F. alocis* stimulates the production of proinflammatory cytokines such as IL-1*β*, IL-6, and TNF-*α* [58]. This is important, since these cytokines are related to the stimulation of osteoclasts and bone resorption [58]. Moreover, these cytokines have been shown to increase in an *in vivo* model (mouse subcutaneous chamber model) and to increase the influx of neutrophils to the site of infection [59].

In spite of the growing evidence showing the relevance of a number of species in the progression of periodontitis, one of the most studied species is *P. gingivalis*. Through such studies, nowadays, we have a good understanding of its role in the pathogenesis of periodontitis. This bacterium is internalized by macrophages and is also able to induce its own internalization by GECs. Once the bacterium is inside the GECs, it can use the machinery of the host cell for its survival and persistence. For example, infected GECs activate antiapoptotic pathways, such as the JAK/STAT and phosphatidylinositol 3-kinase (PI3K)/Akt, which inhibit the intrinsic pathway of apoptosis probably to persist for longer periods. Both pathways have also been related to inflammation. Some cytokines such as IL-6, TNF-*α*, or IFN-*γ* function through the JAK/STAT pathway [60]; additionally, the JAK/STAT pathway activates NF-*κ*B and stimulates TNF-*α* production [61]. The PI3K/Akt pathway, on the other hand, is involved in the increase of TLR4 mRNA, in response to bacterial lipopolysaccharide (LPS) [62]. Finally, phosphorylation of Akt and its consequent activation induces NF-*κ*B, which increases the transcription of antiapoptotic genes [63].

Periodontitis-associated species seek to prolong bacterial growth within the infected cell and also evade the immune system. Once *P. gingivalis* is internalized, it is incorporated into early phagosomes, where it prevents fusion to the lysosome and therefore its degradation [64]. *P. gingivalis* secretes the nucleoside diphosphate kinase (NDK) enzyme that removes ATP through the P2X7 receptor. In macrophages, this receptor stimulates the production and secretion of IL-1*β*, the apoptosis of the host cell, and killing of bacteria [65].

Moreover, infection of human monocytic cell line with *P. gingivalis* activates NLRP3 and AIM2 inflammasome through caspase 1 activation, which produces the processing of pro-IL-1*β* to its active form IL-1*β* [66]. During periodontitis progression, tissue damage occurs both by the direct effect of bacterial virulence factors and the deregulation of the immune system response. *P. gingivalis* interacts with the GECs through the TLRs mediated by the recognition of *P. gingivalis* virulence factors such as fimbria and the LPS. It has been shown that this interaction increases the transcription of TLR2 and TLR4 in GECs [67]. Intriguingly, *P. gingivalis* can modify the lipid A region of its LPS by incorporating different units of acyl groups to its structure. A tetra-acylated structure of *P. gingivalis* lipid A is a TLR4 antagonist with anti-inflammatory potential [68]. However, the penta-acylated structure of *P. gingivalis* lipid A is a TLR4 agonist with proinflammatory potential [68] that activates the NF-*κ*B and MAPK-p38 pathways [69]. Nevertheless, *P. gingivalis* has developed strategies to evade or delay the immune response. For example, within its virulence factors, it possesses gingipain proteases that degrade the CD14 protein (a coreceptor of TLR4 and TLR2), interfering with the optimal recognition of bacterial LPS [70].

*P. gingivalis* can also modify the expression of adhesion receptors—like E-selectin—for leukocyte adhesion and transmigration, preventing its upregulation. In this context, gingipain proteases produced by *P. gingivalis* degrade the intracellular adhesion molecule 1 (ICAM-1) in GECs, disrupting neutrophils-oral epithelial cell interaction [71]. These proteases affect also the integrity of the cytokines IL-6, IL-8, IL-12, and TNF-*α*, which are produced in response to the infection [72–75].

Interestingly, in addition to the inflammation induced by periodontitis-associated bacteria, some “core species” have also been linked to inflammation. For example, it has been demonstrated that *F. nucleatum* upregulates the production of MMP-13 and IL-8, through the MAPK/p38 pathway in epithelial cells [76]. Moreover, *F. nucleatum* increases IL-8 mRNA levels through the activation of NF-*κ*B in human GECs [77].

Similar to *P. gingivalis*, *F. nucleatum* also activates NLRP3 inflammasome, inducing the releases of damage-associated molecular patterns (DAMPs) like high mobility group box 1 protein (HMGB1) and proteins that recruit and activate caspases (ASC), increasing the inflammation in GECs [78]. After infection, HMGB1 is released into the extracellular space, which is required for the activation of the inflammasome and the caspase 1 activation [79, 80]. On the other hand, ASC functions as an adapter of the NLRP3 inflammasome assembly and is secreted by macrophages during inflammation [81].

Limited data exist regarding the effect of combined subgingival species in carcinogenesis. Coinfection studies using *F. nucleatum* and *P. gingivalis* show that they induce a synergic virulence response in a mouse periodontitis model, with a stronger inflammatory response triggered by elevated levels of TNF-*α*, NF-*κ*B, and interleukin IL-1*β* [82], as well as higher levels of attachment and invasion into host cells [83, 84].

## 3. Systemic Diseases Associated with Chronic Inflammation in Periodontitis

Although the inflammatory processes occur locally in the oral cavity, several studies have determined that the chronic inflammation during periodontal diseases or the dissemination of bacterial components could cause various extraoral diseases. Some of these diseases and a brief description of their associations with periodontal disease are summarized as follows:

- *Cardiovascular diseases*: many studies have linked the presence of periodontal diseases with cardiovascular diseases [5, 85, 86]. Among them, Peng et al. [86] determined through a retrospective cohort study that periodontal therapy promoted a decreased risk of cardiovascular disease. Also, different meta-analyses have managed to link the presence of periodontal diseases with an increased risk of cardiovascular disease [85, 87]. Moreover, some periodontitis-associated species have been linked to such diseases. Thus, Damgaard et al. [88] linked the presence of IgG antibodies against *P. gingivalis* with the presence of cardiovascular disease in serum from 576 participants and Bale et al. [89] proposed that *A. actinomycetemcomitans*, *P. gingivalis*, *T. forsythia*, *T. denticola*, and *F. nucleatum* are related to higher risk of atherosclerosis. Interestingly, some cardiovascular diseases are related to chronic inflammation. Two of them, myocarditis and endocarditis, are diseases characterized by a high infiltration of lymphocytes and monocytes. *P. gingivalis* is proposed as an aggravator of autoimmune myocarditis in an *in vivo* model [90].
- *Rheumatoid arthritis (RA)*: RA is an autoimmune disease characterized by the thickening of the synovium, a tissue that exists inside the joints. IL-1 and TNF-*α* are highly related with the pathogenesis of RA, but other cytokines like IL-4 and IL-17 have also a role in this disease. Many studies confirm a relationship between periodontitis and RA [4, 91], like Mikuls et al. [92] who were able to determine that periodontitis and the presence of *P. gingivalis* is related to the self-activity, characteristic of RA. Additionally, both *F. nucleatum* and *P. gingivalis* are highly prevalent in patients with RA [93].
- *Cancer*: it has been shown that patients affected by periodontal disease have a higher risk of suffering from some type of cancer [34]; specifically, a positive association between periodontal disease and orodigestive cancers (oral, esophageal, gastric, colonic, and pancreatic) has been well established [2, 3], as well as other types of cancers such as breast, prostate, and bladder [48, 94–96]. A deeper explanation of such associations and possible mechanisms involved in these associations will be addressed in following paragraphs.

## 4. Association between Periodontitis and Orodigestive Cancer

As stated above, multiple epidemiological studies showed a strong association between orodigestive cancers and poor oral health [97–102], periodontal diseases [103–106], tooth loss [98, 99, 101, 102, 106, 107], and periodontal diagnostic parameters such as clinical attachment loss (CAL) and alveolar bone loss [108, 109]. Additionally, patients showing gastric precancerous lesions were more likely to have higher percentages of sites with gingival bleeding [97, 110].

**Figure 1:**
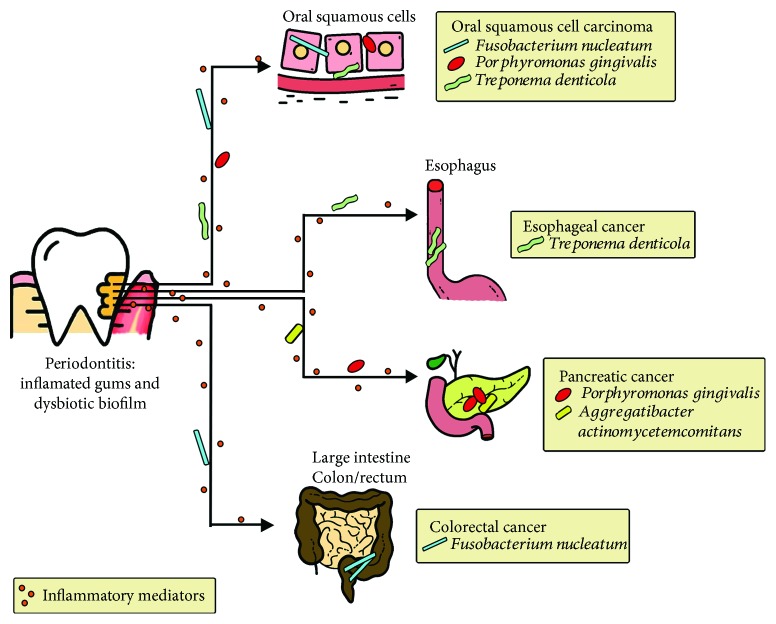
Association of periodontal bacteria with orodigestive cancer. Periodontitis has been associated with orodigestive cancers through the chronic inflammation generated in the oral cavity and the concomitant mobilization of inflammatory mediators to distal sites in the human body, as well as a direct carcinogenic effect mediated by periodontitis-associated bacterial species either directly in oral cells or by migrating from the oral cavity.

Together with the increasing evidence associating periodontal diseases with several types of extra oral cancer, the question of how these bacteria exert their effect in distal sites in the human body is gaining more and more attention. Thus, some types of cancer have associated carcinogenesis with the chronic inflammation generated in the oral cavity and the concomitant mobilization of inflammatory mediators to distal sites in the human body (Figure 1) [3, 111], while other studies have associated it with a direct carcinogenic effect mediated by periodontitis-associated bacterial species either directly in oral cells or by migrating from the oral cavity (Figure 1) [112]. Interestingly, despite the natural dissemination of oral bacteria due to swallowing of saliva, which contains a large number of bacteria, explaining therefore its involvement in orodigestive tract [113, 114], there is also evidence showing dissemination through the bloodstream (Figure 1) [115].

Systemic spread of oral bacteria either after routine activities or dental procedures was early reported by Cobe [116]. Particularly, oral anaerobes are released to circulation after some daily activities, such as tooth brushing, flossing, and chewing [117], and also immediately after therapeutic oral procedures such as scaling and root planning [118]. Therefore, dental or oral surgery is considered to be a predisposing factor for anaerobes bacteremia in both adults and children [119, 120]. However, in periodontal disease, migration of bacteria from the oral cavity to other organs in the human body is likely to occur through the blood circulation probably because there is a 3-log increase in the biomass of the subgingival biofilm and the mean surface area where this biofilm is contacting the ulcerated gingiva is approximately 20 cm^2^ [1, 121], providing a portal of entry for oral bacteria into the vessels and thereby allowing them to spread to distant sites [122]. These bacteremias are usually polymicrobial, with higher numbers of Gram-negative bacilli and species of the genera *Peptostreptococcus*, *Clostridium*, *Fusobacterium*, among others [115].

As stated above, *F. nucleatum* is part of the subgingival microbiota and it is present in most subjects maintaining its proportion from health to disease, probably acting as a metabolic cornerstone for the whole community. Interestingly, extensive evidences associating bacteremia caused by *F. nucleatum* with underlying malignancy have been reported [123]. Moreover, comorbidity between *F. nucleatum* bacteremia and several types of cancer has been found in hospitalized patients [124–126]. Particularly, *F. nucleatum* is considered as a risk factor for colorectal cancer (CRC) (Figure 1) [7, 127, 128], as the bacterium is overrepresented in colorectal tumor tissues versus normal tissues in CRC patients [129–131]; moreover, higher loads of the bacterium have been found in CRC compared to premalignant lesions [127]. It is worth noting that as the bacterium is found together with other oral species in CRC such as *Parvimonas micra*, *Peptostreptococcus stomatis*, *Gemella morbillorum*, *Porphyromonas* spp, *Leptotrichia* spp., and *Campylobacter* spp., it strongly suggests that the source of the microbes is the oral cavity [130, 132–135]. More recently, *F. nucleatum* was also associated with other malignancies as oral cancer (Figure 1) [7], with higher levels of this species found in oral squamous cell carcinoma (OSCC) patients compared to controls [136, 137]. Similar to CRC, other periodontitis-associated taxa, such as *Dialister* spp., *Peptostreptococcus* spp., *Filifactor* spp., *Treponema* spp., and *Parvimonas* spp., were also enriched in these tumors [138]. This is interesting since a combined effect of such species could contribute to cell transformation.

Remarkably, the periodontitis-associated species *P. gingivalis* is the oral bacteria most commonly associated with cancers of the orodigestive tract and it probably has a positive effect in mortality [6, 139]. Among these cancers, *P. gingivalis* shows a strong correlation with OSCC [136], as well as with pancreatic cancer (Figure 1) [6, 140]. This species has been found in tumor tissues from patients with OSCC along with other oral anaerobes as species of the genera *Veillonella*, *Fusobacterium*, *Prevotella*, *Actinomyces*, and *Clostridium* [141], indicating that a combined effect of multiple bacterial species may be involved in carcinogenesis. Similar results have been observed in gingival squamous cell carcinoma where *P. gingivalis* is augmented compared to normal tissues [142], probably due to its invasive ability. In fact, tissue invasion is probably one of the significant ways of oral bacteria dissemination, since both *F. nucleatum* and *P. gingivalis*—the oral species mostly associated with orodigestive cancers—invade gingival tissues and have been found composing 15% to 40% of the total bacteria within the gingival tissue obtained from periodontal lesions [143]. The occurrence of both species in the tissue is likely to happen as a consequence of an intimate interaction between them in the oral cavity and probably also in extra oral sites.

Remarkably, the sole presence of a bacterium in tumorous tissue is not necessarily indicative of its role in the disease. A recent metatranscriptomic analysis showed that although both *P. gingivalis* and *F. nucleatum* were active in OSCC tumor sites compared to healthy control tumor-matched sites, only *F. nucleatum* showed a significant difference in transcriptional activity, as shown by linear discriminant analysis effect size (LefSe) analysis [144]. This indicates that either different species have a role in different stages of the tumorigenesis or that close interactions between microbial species in the tumoral tissues may modify the gene expression of the companions, as it has been shown in an *in vitro* multispecies community model [145, 146]. Interestingly, although it is not a periodontitis-associated species, *F. nucleatum* has been found to be transcriptionally active in different forms of periodontal diseases [147, 148]. Moreover, synergistic interactions between *F. nucleatum* and two periodontitis-associated bacteria, *T. denticola* and *P. gingivalis*, have been reported in chronic periodontitis [149].

This is interesting, since in addition to *P. gingivalis*, other periodontitis-associated taxa have been associated with orodigestive cancers. While carriage of *A. actinomycetemcomitans* correlates with higher risk of pancreatic cancer [150], *T. denticola* has been detected in both tongue squamous cell carcinoma [151] and esophageal cancer tissues (Figure 1) [152]. The question of how these species interact with each other in carcinogenesis has not been fully understood. It has neither been elucidated how migrating oral bacteria affect the local microbiome in distal sites and therefore alter host cell responses. For instance, Arimatsu et al. [153] showed that oral administration of *P. gingivalis* induces changes in the ileal microbiota in a mouse model, increasing systemic inflammation.

## 5. The Mechanism of Cancer Promotion by Periodontitis-Associated Bacteria

Although the exact mechanisms involved in cancer promotion by periodontal bacteria have not been completely elucidated, local inflammatory effects triggered by bacterial infection have been associated with cellular transformation [6]. Moreover, among all the subgingival species found in tumorous tissue, there is only information regarding carcinogenic mechanisms triggered by a few of them.

**Figure 2:**
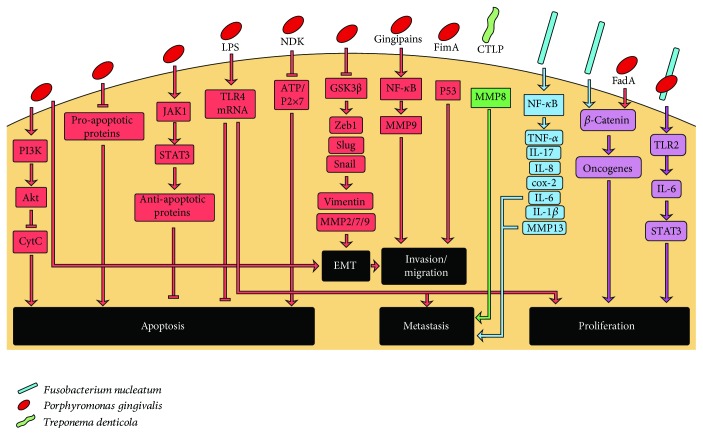
Host response mechanisms of cellular transformation induced by periodontal bacteria. Inhibition of apoptosis, epithelial-mesenchymal transition (EMT), invasion and migration, metastasis, and proliferation are triggered through the activation of prooncogenic pathways by *P. gingivalis* (red arrows), *T. denticola* (purple arrows), *F. nucleatum* (yellow arrow), and *P. gingivalis*+*F. nucleatum* coinfection (orange arrows).

*P. gingivalis* was shown to activate carcinogenesis through several mechanisms (Figure 2). First, the bacterium has been associated directly with activation of oncogenic pathways, such as the promotion of survival in GECs through both the activation of the PI3K/Akt pathway and the inhibition of cytochrome c release [11], as well as with the reduction of the expression of proapoptotic proteins [10]. Additionally, *P. gingivalis* blocks apoptosis through the JAK/STAT pathway in GECs and therefore modulates the intrinsic cell death pathway and regulates the expression of several antiapoptotic proteins [154]. The LPS of *P. gingivalis*, in particular the O-antigen region, contributes to the apoptosis inhibition and induces proliferation in GECs [67]. This effect is associated with increased expression of TLR4 [67].

*P. gingivalis* was also shown to induce GECs migration in a manner dependent on the overexpression of Zeb1 [155], an activator of the epithelial-mesenchymal transition (EMT). Moreover, *P. gingivalis* increases proliferation and promotes invasion and migration in an *in vitro* model of persistent infection [9]. Likewise, *P. gingivalis* infection inhibits the activity of glycogen synthase kinase 3 (GSK3b), an important EMT regulator, in primary oral epithelial cells [156]. Additionally, other EMT-associated transcription factors, as well as mesenchymal intermediates, such as vimentin, MMP-2, MMP-7, and MMP-9, are increased and associated with higher levels of cell migration.

Several virulence factors are involved in the direct activation of inflammation and cell proliferation mediated by *P. gingivalis* [6]. Among them, nucleoside diphosphate kinase (NDK), FimA, and the LPS of *P. gingivalis* participate in the first stages of carcinogenesis, while gingipains and GroEL are associated with later stages. NDK inhibits proapoptotic mechanisms in oral epithelial cells by inhibiting the ATP/P2X_7_ cell death signaling [65, 157, 158]. FimA attenuates the host p53-mediated tumor suppression and cell cycle progression in oral epithelial cells [6, 67] and controls the epithelial–mesenchymal transition [155]. Gingipain proteases of *P. gingivalis* activate NF-*κ*B and MMP-9 in oral squamous carcinoma cells, which is important for cancer cell invasion and metastasis [159, 160]. Finally, GroEL produced by *P. gingivalis* increases tumor volume and the mortality of mice implanted with the mouse colon carcinoma cell line (C26) [161]. Recently, Mfa1 fimbria was shown to induce oncogenic signaling, producing myeloid-derived dendritic suppressor cells (MDDSCs) from monocytes activating the pAKT1-pFOXO1 pathway through dendritic cell-specific intercellular adhesion molecule 3-grabbing non-integrin (DC-SIGN) receptor [162].

Although comparatively less information exists regarding carcinogenic mechanisms triggered by *F. nucleatum*, three virulence factors have been associated with CRC promotion: the adhesin FadA, the LPS, and the autotransporter protein Fap2 (Figure 2) [7]. FadA induces inflammation and activation of procarcinogenic pathways directly in colorectal cells, activating E-cadherin-*β*-catenin signaling [163]. The LPS of *F. nucleatum* induces the production of inflammatory cytokines both in the gingiva and in the colonic tissue [129, 164]. Consistently, increased expression of proinflammatory cytokine such as IL-6, IL-12, IL-17, and TNF-*α* has been found in *F. nucleatum*-enriched colorectal adenoma subjects compared to nonadenoma controls [165]. Finally, Fap2 decreases the cytotoxicity of immune cells, favoring cancer progression [166]. *In vivo* studies showed that *F. nucleatum* increases tumor multiplicity and recruitment of tumor-infiltrating immune cells in a mouse model of intestinal tumorigenesis [167]. In this model, *F. nucleatum* generates a proinflammatory microenvironment associated with an NF-*κ*B-mediated response (COX-2, IL-1*β*, IL-6, IL-8, IL-10, and TNF-*α*) [167], which provides a critical link between inflammation and cancer [168] and is implicated in potentiating colorectal tumorigenesis in mice [167, 169]. In addition, *F. nucleatum* increases the proliferation and invasion ability of colonic epithelial cells, promoting EMT, activating NF-*κ*B signaling, and increasing the production of IL-6, IL-1*β*, and MMP-13 [170].

Even less studies evaluated the association of other periodontitis-associated taxa with cancer, among them the contribution of *T. denticola* to carcinogenesis has recently been reported (Figure 2). This species is a highly invasive anaerobic bacteria and possesses a chymotrypsin-like proteinase (CTLP) as a major virulence factor. Recently, CTLP was detected within orodigestive tumor tissues including OSCC, tongue, tonsil, and esophagus [171]. Intriguingly, CTLP converts pro-MMP-8 and pro-MMP-9 to their active forms, which are associated with metastasis in tongue, esophageal, gastric, pancreatic, and CRC [8, 12, 172].

As mentioned above, systemic spread of periodontitis-associated bacteria is usually polymicrobial. In this context, although combined effect of periodontal bacteria is well established in the etiology of periodontitis, its contribution to cancer onset is less understood. Therefore, it is relevant to understand if these bacterial cooccurrences have synergistic or antagonist effect in respect to the activation of inflammatory pathways associated to cancer.

In this context, it has been shown that coinfection of oral epithelial cells with *P. gingivalis* and *F. nucleatum* triggers the TLR2 pathway resulting in IL-6 production and STAT3 activation, which in turn stimulate cell proliferation (Figure 2) [173]. In addition, infection of oral epithelial cells with cocultures of *P. gingivalis* and *F. nucleatum* induces a slight increase in cell migration [156]; however, the pathways that are altered and could explain this effect have not been defined.

## 6. Conclusion

Periodontitis is a dysbiotic disease, in which chronic inflammation is produced in response to a disease-associated multispecies bacterial community established in the subgingival area. The recruitment of immune cells and the production of several inflammatory mediators contribute to the tissue damage. Additionally, the direct effect of periodontitis-associated bacteria as well as other subgingival microorganisms equally prevalent both in healthy and diseased subjects “core species” contributes to the chronicity of the disease through the activation of specific inflammatory pathways.

Chronic inflammation has also been associated with several systemic diseases, like cancer. The literature demonstrates that either inflammatory mediators produced during periodontitis development could mediate carcinogenesis or periodontal bacteria can exert its effect directly in transforming cells. Interestingly, several oral bacteria, also found in high loads in the periodontal pocket, have been shown to activate inflammatory pathways associated with several stages of cellular transformation (Figure 2). Among them, these bacteria can induce NF-*κ*B-mediated responses, promote cell survival, activate oncogenic pathways, reduce proapoptotic proteins expression, increase cell migration and invasion, increase the expression of EMT-associated proteins, enhance metastasis, etc. In spite of this knowledge, more studies are needed to elucidate the mechanisms triggered by other periodontal bacteria and also understand the tumorigenic effect of combined bacterial infections. Such studies are relevant because, although the combined effect of species such as *P. gingivalis* and *F. nucleatum* has been studied in the etiology of periodontitis, the consequences of its effect in carcinogenesis remain poorly understood. Moreover, since bacterial spreading to distant sites on the human body occurs in coexistence, it is relevant to know the synergistic or antagonistic effects that these interactions may have in oral and extra oral carcinogenesis.
